# Development and validation of prognostic nomographs for patients with cervical cancer: SEER-based Asian population study

**DOI:** 10.1038/s41598-024-57609-7

**Published:** 2024-04-01

**Authors:** Siyuan Zeng, Ping Yang, Simin Xiao, Lifeng Liu

**Affiliations:** 1 Department of Obstetrics and Gynecology, Dalian Municipal Central Hospital, Dalian, Liaoning China; 2 Dalian Municipal Central Hospital, China Medical University, Shenyang, Liaoning China; 3 Department of Radiation Oncology, the First Affiliated Hospital of Dalian Medical University, Dalian, Liaoning China; 4 Department of Radiology, Chengdu Xindu District Traditional Chinese Medicine Hospital, Chengdu, Sichuan China

**Keywords:** Cervical cancer, Prognosis, Nomogram, SEER, Cancer, Medical research

## Abstract

To develop and validate a nomograph to predict the long-term survival probability of cervical cancer (CC) patients in Asia, Surveillance, Epidemiology, and End Results (SEER) were used to collect information about CC patients in Asia. The patient data were randomly sampled and divided into a training group and a validation group by 7:3. Least absolute shrinkage and selection operator (LASSO) regression was used to screen key indicators, and multivariate Cox regression model was used to establish a prognostic risk prediction model for CC patients. The receiver operating characteristic (ROC) curve and decision curve analysis (DCA) were adopted to comprehensively evaluate the nomogram model. LASSO regression and multivariate Cox proportional hazards model analysis showed that age, American Joint Committee on Cancer (AJCC) Stage, AJCC T, tumor size, and surgery were independent risk factors for prognosis. The ROC curve results proved that the area under curve (AUC) values of the training group in 3 and 5 years were 0.837 and 0.818, The AUC values of the validation group in 3 and 5 years were 0.796 and 0.783. DCA showed that the 3- and 5-year overall survival (OS) nomograms had good clinical potential value. The nomogram model developed in this study can effectively predict the prognosis of Asian patients with CC, and the risk stratification system based on this nomogram prediction model has some clinical value for discriminating high-risk patients.

## Introduction

Globally, cervical cancer (CC) is the third most common cancer among women and the second most common cancer among Asian women^1^, and the number of new cases and deaths worldwide in 2018 was 570,000 and 311,000, respectively^2^. Although the advent of human papillomavirus vaccines is effective in blocking the occurrence and progression of cervical disease, the incidence of CC is slowly increasing in some areas^3^. There are many studies on CC based on Surveillance, Epidemiology, and End Results (SEER) database, while they do not focus on Asian patients only, but also multi-ethnic patients included in SEER. However, many studies specify that tumor prognostic factors are related to ethnicity, while many studies do not have detailed studies on Asian CC patients^4–6^.

Recently, there have been many studies to establish various models affecting prognostic factors to determine the prognosis of CC patients. Federation of International Gynecology and Obstetrics (FIGO) staging is the most used staging system developed based on clinical examination. However, FIGO staging does not involve age, race, and other demographic factors, and it includes patients with the same anatomical spread but different survival outcomes in the same staging, thus leading to heterogeneity of survival outcomes of patients in the same staging, which makes FIGO staging lack relatively accurate individual prediction ability for cervical cancer patients in Asia^7^. The American Joint Commission on cancer (AJCC) has proposed a tumor node metastasis (TNM) staging classification, which states the presence or absence of metastasis in regional lymph nodes and distant organs based on clinical staging before treatment^8,9^. However, the prognosis based on the TNM staging system is still limited and cannot accurately predict prognosis. To develop an individualized treatment plan, it is necessary to consider all the cancer-related risk factors. In this paper, based on the SEER database, Asian CC patients were selected as the research object, and the prognostic impact factors of common CC were studied and analyzed, aiming to guide clinical prognosis judgment and treatment. The novelty lies in our topic of to develop and validate a nomogram to predict the long-term survival probability of CC patients in Asia. The results indicate that the nomogram model developed in this study can effectively predict the diagnosis of Asian patients with CC, And the risk differentiation system based on this nomogram prediction model has some clinical value for discriminating high risk patients, based on the literature we searched for during our submission, there are currently no researchers conducting research on similar topics.

## Methods

### Data source and data extraction

From the SEER database, the data on Asian female patients diagnosed with CC between 2004 and 2015were downloaded. The research methods used in this study seriously followed the research guidelines published in the SEER database. The clinicopathological data of all Asian female CC patients were collected, including age, marital status, FIGO stage, TNM stage, tumor size, surgery, radiotherapy, chemotherapy, lymph node metastatic status, and tumor multiple primary status. SEER was also used to obtain patient follow-up information, including survival status and survival time (Supplementary Fig. 1).

### Inclusion and exclusion criteria

Inclusion criteria: (1) histopathological diagnosis of primary CC based on the international classification of diseases, the third edition (ICD-O-3), and patients with c53.0, c53.1, c53.8, and c53.9 between 2004 and 2015, (2) the primary site of tumor was cervical, (3) clinical information was accurate and reliable. Exclusion criteria: (1) non-Asian population, (2) patients with incomplete tumor information or treatment information and patients with a survival time of less than 1 month. Finally, 1567 patients were included in this study (Supplementary Table 1).

### Statistical analysis

The R 4.1.2 software was used for statistical analysis and modeling, the “caret” package was used to randomly divide the training and validation groups by a ratio of 7:3, and the intergroup comparison of count data was performed using the χ^2^ test. Least absolute shrinkage and selection operator (LASSO) regression was performed with the “glmnet” package to screen out the meaningful indexes that were used to build a Cox regression model and construct a nomogram. The discrimination of the model was evaluated in terms of the receiver operating characteristic curve (ROC) and area under curve (AUC), respectively; calibration curves assessed the model consistency and decision curve analysis (DCA) assessed the clinical validity of the model. The risk of death was calculated for each patient based on the nomogram, and the optimal cut off value was found based on the time-dependent ROC curve, which divided the patients into high and low-risk groups. Survival analysis was performed using the Kaplan–Meier curves for high-risk and low-risk groups, separately, and log rank was used to compare differences. *P* < 0.05 was regarded to be statistically significant.

## Results

### Clinical features

According to the inclusion criteria, the total number of cases finally included in this study was 1567. 1097 patients in the training group and 470 patients in the validation group. The percentages were 41.16% for those aged < 48 years, 47.67% for those aged 48–71 years, 11.17% for those aged ≥ 72 years. According to the best cut-off point of K-M curve, the age and the tumor size was divided into three categories, and the tool used is X-tile (Supplementary Figs. 2, 3). Table 1 showed the training set and validation set divided randomly and hierarchically. The training set was used for modeling and evaluation, and the validation set was used for reevaluation of the model. The validation results of the validation set were convincing only when there were no differences between the baseline tables of the training set and the validation set. A total of 60.11% among those were married, and 39.89% among those were single. There were no statistical differences in any of the indicators between the two groups (*P* > 0.05). In this study, married people are defined as a category (frequent sexual life), and the rest of unmarried, divorced and other unmarried people are classified as single (infrequent sexual life). The purpose was to compare the two categories of people.

**Table 1:** Clinicopathological characteristics of CC patients in Asia.

Variable	All (n = 1567)	Training set (n = 1097)	Validation set(n = 470)	*P*
Age				0.733
< 48	645 (41.16)	449 (40.93)	196 (41.70)	
48–71	747 (47.67)	521 (47.49)	226 (48.09)	
≥ 72	175 (11.17)	127 (11.58)	48 (10.21)	
Marital_status				0.365
Single	625 (39.89)	429 (39.11)	196 (41.70)	
Married	942 (60.11)	668 (60.89)	274 (58.30)	
Histological_type				0.272
Squamous cell carcinoma	987 (62.99)	701 (63.90)	286 (60.85)	
Adenocarcinoma	414 (26.42)	277 (25.25)	137 (29.15)	
Other	166 (10.59)	119 (10.85)	47 (10.00)	
Grade				0.529
I–II	893 (56.99)	619 (56.43)	274 (58.30)	
III–IV	674 (43.01)	478 (43.57)	196 (41.70)	
AJCC_Stage				0.320
I	771 (49.20)	535 (48.77)	236 (50.21)	
II	266 (16.98)	178 (16.23)	88 (18.72)	
III	377 (24.06)	277 (25.25)	100 (21.28)	
IV	153 ( 9.76)	107 ( 9.75)	46 ( 9.79)	
AJCC_T				0.265
T1	931 (59.41)	653 (59.53)	278 (59.15)	
T2	404 (25.78)	273 (24.89)	131 (27.87)	
T3–T4	232 (14.81)	171 (15.59)	61 (12.98)	
AJCC_N				0.196
N0	1185 (75.62)	819 (74.66)	366 (77.87)	
N1	382 (24.38)	278 (25.34)	104 (22.13)	
AJCC_M				0.986
M0	1435 (91.58)	1004 (91.52)	431 (91.70)	
M1	132 ( 8.42)	93 ( 8.48)	39 ( 8.30)	
Tumor_size				0.593
< 2.9	629 (40.14)	432 (39.38)	197 (41.91)	
2.9–5.3	532 (33.95)	380 (34.64)	152 (32.34)	
≥ 5.4	406 (25.91)	285 (25.98)	121 (25.74)	
Surgery				0.085
No	490 (31.27)	358 (32.63)	132 (28.09)	
Yes	1077 (68.73)	739 (67.37)	338 (71.91)	
Radiation				0.995
No	622 (39.69)	436 (39.74)	186 (39.57)	
Yes	945 (60.31)	661 (60.26)	284 (60.43)	
Chemotherapy				0.297
No/Unknown	747 (47.67)	513 (46.76)	234 (49.79)	
Yes	820 (52.33)	584 (53.24)	236 (50.21)	
lymph_node_metastasis				0.196
No	1185 (75.62)	819 (74.66)	366 (77.87)	
Yes	382 (24.38)	278 (25.34)	104 (22.13)	
Multiple_primary				0.570
No	1370 (87.43)	963 (87.78)	407 (86.60)	
Yes	197 (12.57)	134 (12.22)	63 (13.40)	

### Identification of independent prognostic factors for the model and Multivariate COX Regression Analysis

**Figure 1:**
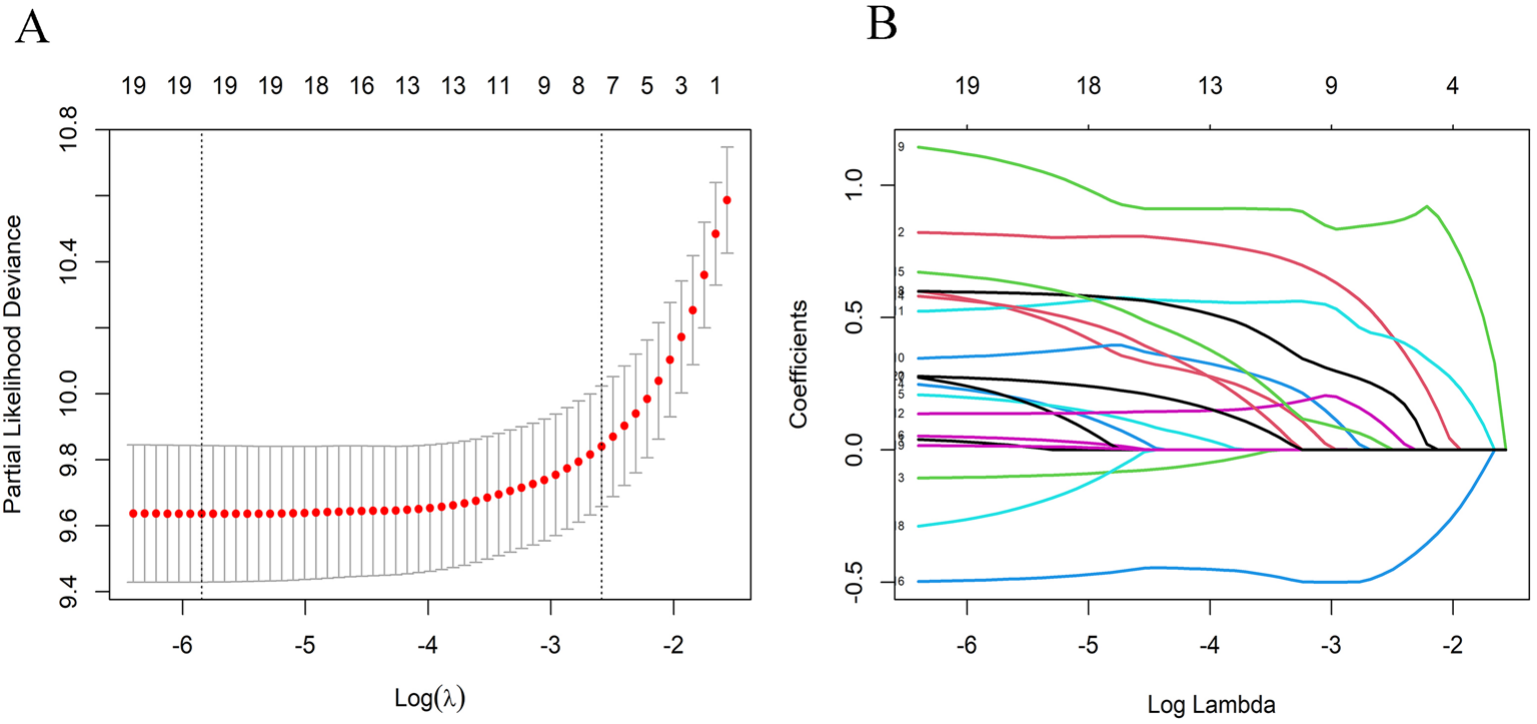
LASSO regression model was used to select characteristic impact factors. (**A**) Selection of tuning parameter (λ) in the LASSO regression using ten-fold cross-validation via 1-SE criteria. (**B**) A coefficient profile plot was created against the log (lambda) sequence. In the present study, predictors selection was according to the 1-SE criteria (right dotted line), where 7 nonzero coefficients were selected. LASSO, least absolute shrinkage, and selection operator; SE, standard error.

**Figure 2:**
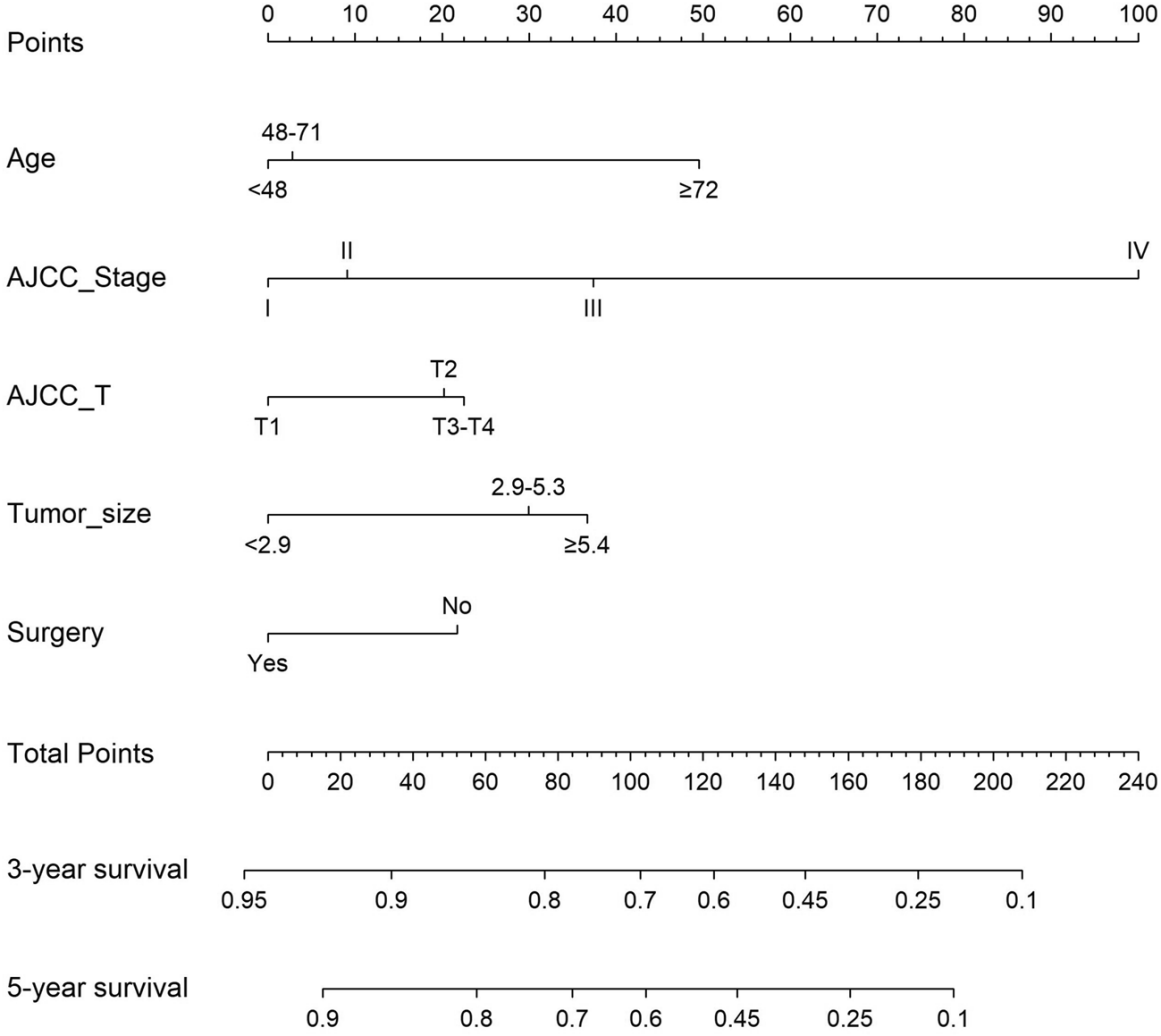
Nomogram including age, AJCC stage, AJCC T, tumor size, and surgery, for three- and five-years OS in Asian patients with CC.

In this study, from 14 variables that may affect the prognostic risk of CC in Asia, 7 meaningful variables were screened using LASSO regression, including age, AJCC stage, AJCC T, AJCC N, AJCC M, tumor size, and surgery. (Fig. 1). The meaningful 7 variables selected by LASSO regression were subjected to multivariate Cox regression analysis, which showed that the 5 variables of age, AJCC stage, AJCC T, tumor size, and surgery were independent risk factors for prognosis. (Table2). By plotting the nomogram with five independent risk factors affecting CC patients in Asia (Fig. 2), the 3- and 5-year overall survival (OS) rates of CC patients in Asia could be predicted visually.

**Table 2:** Multifactor Cox regression results.

Variable	HR	95%CI	*P*
Age			
< 48	1		
48–71	1.053	0.812–1.364	0.698
≥ 72	2.491	1.825–3.401	< 0.001
AJCC stage			
I	1		
II	1.174	0.680–2.027	0.564
III	1.735	1.046–2.877	0.033
IV	3.255	1.515–6.995	0.002
AJCC T			
T1	1		
T2	1.424	0.939–2.161	0.096
T3–T4	1.651	1.092–2.496	0.018
AJCC N			
N0	1		
N1	1.094	0.779–1.535	0.604
AJCC M			
M0	1		
M1	1.784	0.910–3.500	0.092
Tumor size			
< 2.9	1		
2.9–5.3	1.734	1.242–2.421	0.001
≥ 5.4	1.881	1.312–2.698	0.001
Surgery			
No	1		
Yes	0.677	0.524–0.876	0.003

### Construction of a prognostic long term survival nomogram

Multivariate results showed that the five variables of age, AJCC stage, AJCC T, tumor size, and surgery were independent risk factors for prognosis, and the final model was based on the five variables, which were presented as nomograms (Fig. 2). For example, the five indicators of a certain patient are age 60, AJCC stage II cervical cancer, tumor diameter of 2 cm, T1. This patient has undergone surgical treatment, with a total score of 12.5 points. The predicted 3-year survival rate is 92%, and the 5-year survival rate is 90%.

### Validation of nomograms

**Figure 3:**
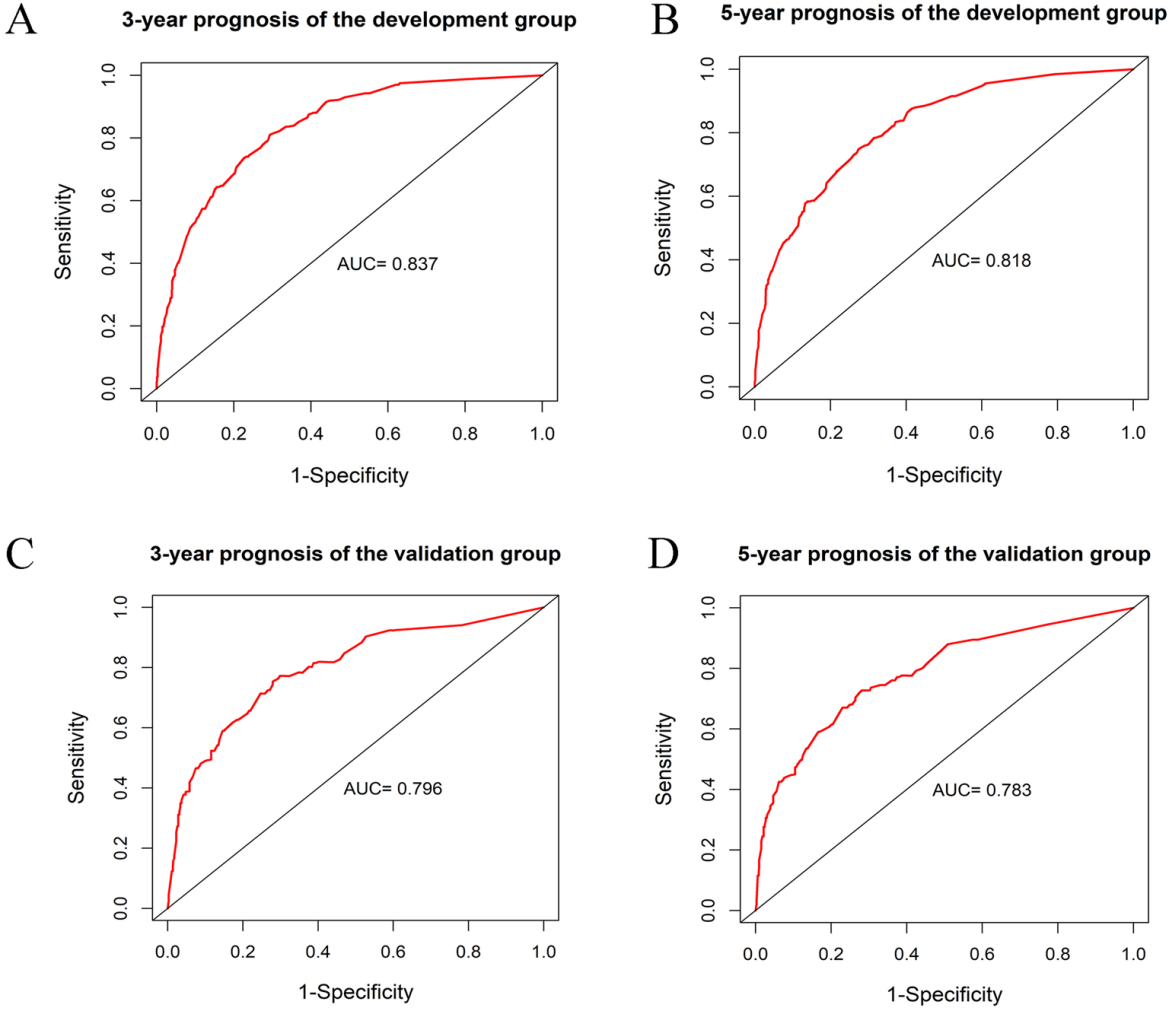
The ROC curve for predicting patient survival at (**A**) 3 years and (**B**) 5 years in the training set and at (**C**) 3 years and (**D**) 5 years in the validation set. The false positive (FP) rate is plotted on the X-axis, and the true positive (TP) rate is plotted on the Y-axis.

**Figure 4:**
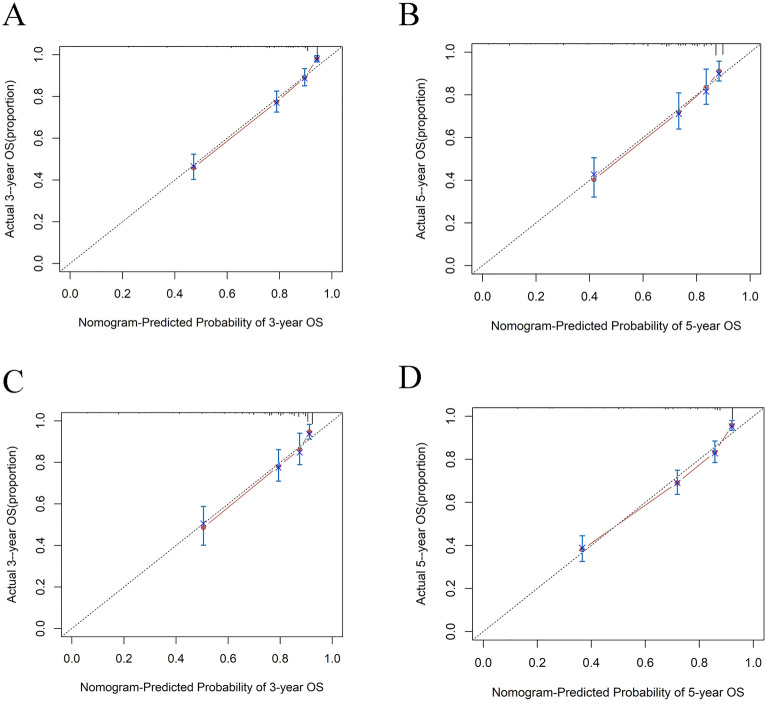
The calibration curve for predicting patient survival at (**A**) 3 years and (**B**) 5 years in the training set, and at (**C**) 3 years and (**D**) 5 years in the validation set. The nomogram-predicted probability of OS is plotted on the X-axis, the actual overall survival is plotted on the Y-axis.

The nomogram was validated with an internal validation method, and the measures were ROC curves and calibration curves. The ROC curve results proved that the AUC values of the training group at 3 and 5 years were 0.837 (95% CI: 0.790–0.882) and 0.818 (95% CI: 0.776–0.856), respectively, while the AUC values of the validation group at 3 and 5 years were 0.796 (95% CI: 0.731–0.858) and 0.783 (95% CI: 0.720–0.853), respectively, which suggested that the nomogram model had good discrimination (Fig. 3). Calibration plot visually displayed the nomogram tumor and predicted probability values versus the actual probability values. As the graph shows, the horizontal axis represents the nomogram's prediction of survival probability for each patient, and the vertical axis shows the actual survival rate, which indicates the ideality when the red line exactly coincides with the black dotted line. According to calibration curves from the present study, the nomogram predicted survival probabilities were in good agreement with the actual survival results (Fig. 4).

### Clinical application of the nomograms

**Figure 5:**
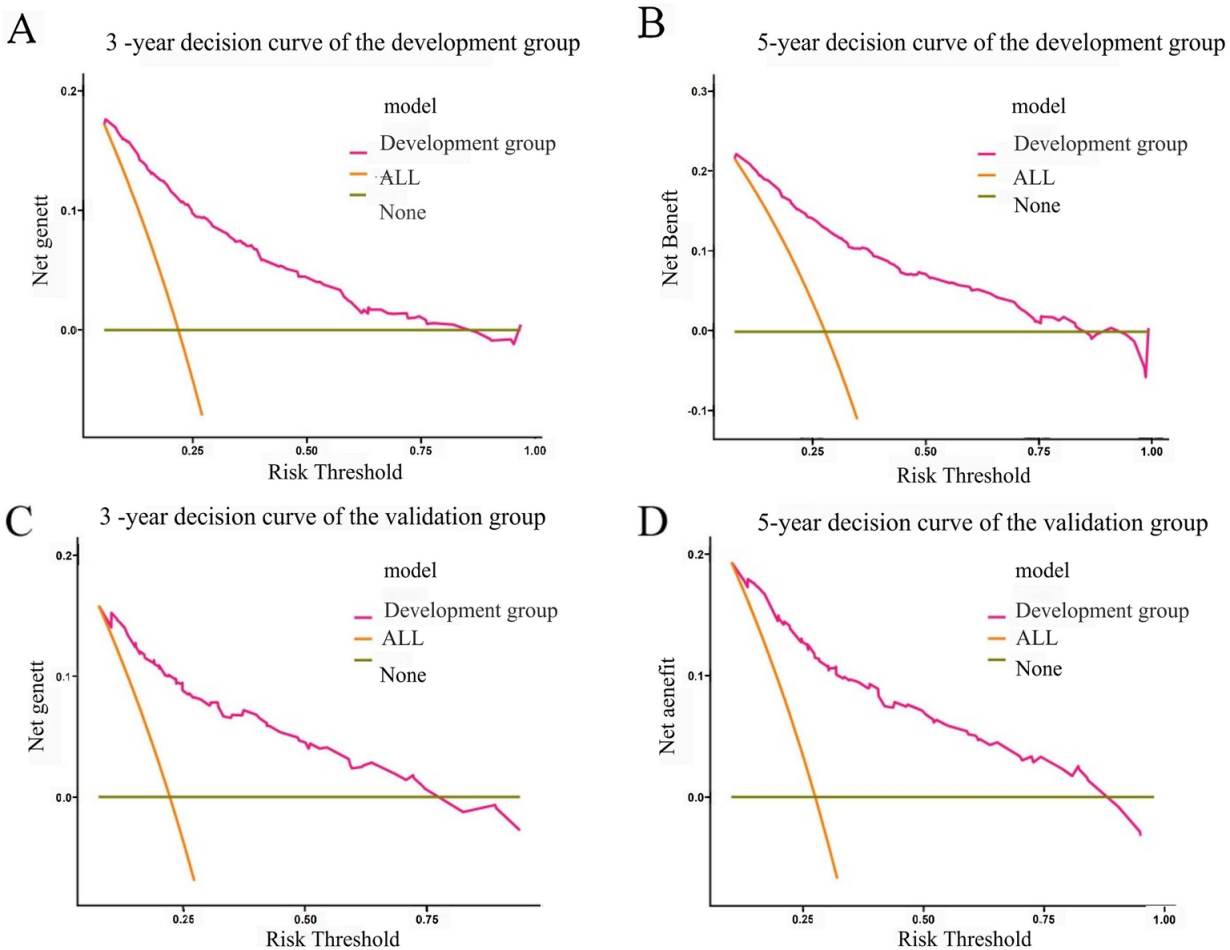
Prognostic decision curve analysis (DCA) of Asian patients with CC. (**A**) 3-year survival DCA of the training set; (**B**) 5-year survival DCA of the training set; (**C**) 3-year survival DCA of the validation set; (**D**) 5-year survival DCA of the validation set.

**Figure 6:**
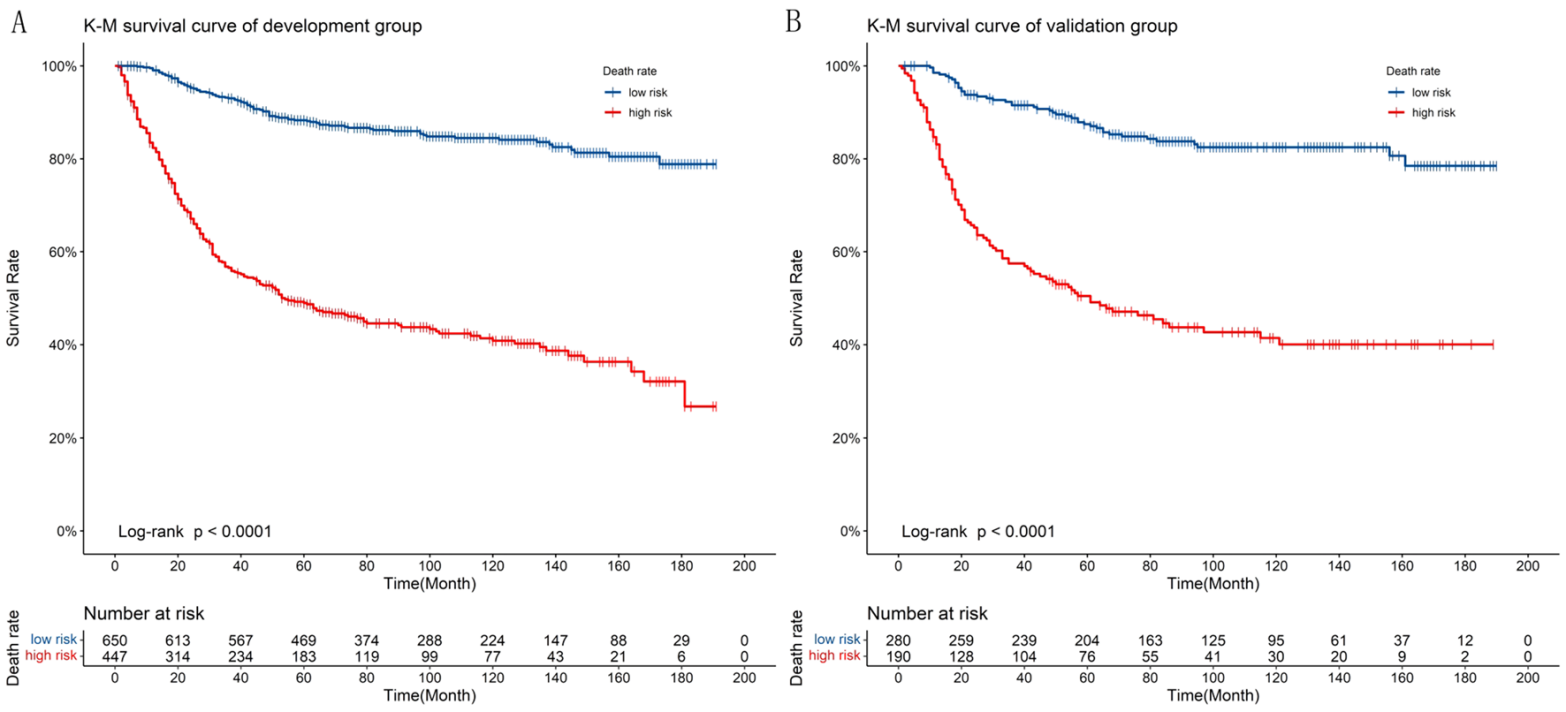
Kaplan Meier curve of patients in low-risk group and high-risk group. K-M curve shows that the incidence of OS in the high-risk group is significantly lower than that in the low-risk group in the training set (**A**), and in the validation set (**B**).

DCA of Asian female patients with CC and Risk stratification: As can be seen from the DCA curves, for both the training and validation cohorts, the predicted probability thresholds of the nomogram for 3 and 5 year OS were in a large range, and the patients all had better clinical net benefits, which indicated that the nomogram model had good clinical applicability (Fig. 5). A risk score was derived for each variable based on the nomogram, and a death probability was calculated for all patients. According to the optimal cut-off value of the ROC curve, the training and validation groups were divided into low- and high-risk groups, respectively. A significant difference (*P* < 0.001) in OS between low and high-risk groups was observed according to the survival curves (Fig. 6). Consequently, the nomogram was able to stratify risks more effectively. For this study, the critical points at different time points are relatively close, and the high and low risk groups divided are completely consistent, so the K-M curve is also completely consistent. Only one result needs to be presented, and there is no need to distinguish between 3 years and 5 years.

## Discussion

The application of computational biology methods to analyze and predict the prognosis of tumors and establish prognostic models is gradually becoming a routine method in tumor research. For example, an ODE-based theoretical modeling of gene/protein signaling networks studies NLRP1B inflammasome signaling. Biochemical reactions in the NLRP1B inflammasome signaling pathway are represented by molecular-molecular interactions and enzymatic reactions, the rate of which depends on the amount of protein and the kinetic rate constant according to the law of mass action. This model can well describe the death state controlled by Caspase-1 or GSDMD in single cells^10^. The receiver operating characteristic (ROC) curve is an important model for evaluating binary classification problems in the bioinformatics. The ROC curve can be drawn under plane rectangular coordinate system. The true positive rate (TPR) was taken as the horizontal coordinate and the false positive rate (FPR) as the vertical coordinate. The area under the curve is AUC^11^. In addition, artificial intelligence plays an important role in medical research. A new deep learning algorithm, called graph convolutional network with graph attention network (GCNAT), is proposed to predict the potential association of disease-related metabolites^12^. Another research project proposed a method for predicting human lncRNA-miRNA interactions based on Graph Convolutional Neural Networks (GCN) and Conditional Random Fields (CRF), named GCNCRF^12^.

The nomogram is one statistical model for determining the quantitative relationship between multiple risk factors and tumorigenesis and (or) prognosis, which enables the prediction value of each outcome event to be calculated, thus transforming complex regression equations into a visual representation, enhancing the readability of prediction model results and making patient evaluation easier. At present, many studies have constructed precise nomograms to predict the long-term prognosis of CC patients, and these models can help clinicians design personalized treatments for CC patients^13–20^. However, a common limitation of these studies is that the study subjects did not target Asian patients, and it is widely recognized that genetic differences among different ethnicities are also important risk factors for tumor prognosis^4,17,19,21,22^. A study on cervical squamous cell carcinoma (associated with HPV infection) revealed the same prognostic indicators and conservative differentiation in two different subgroups. This study conducted a comprehensive multi-component analysis of 643 cases of cervical squamous cell carcinoma (CSCC). As the most common histological variant of cervical cancer, it represents the patient population from the United States, Europe and sub Saharan Africa, and identified two CSCC subtypes with different prognosis. The results of the two subgroups were different in the three continents, and the new development of this knowledge may affect the further analysis of prognosis indicators of cervical cancer patients^23^.

According to the World Health Organization (WHO), CC incidence ranks second among female genital malignancies, and there are 530,000 new cases and approximately 250,000 female factor CC deaths annually worldwide. In this paper, data were extracted from the SEER database, and the total number of cases finally included in this study was 1567 patients, with 1097 patients in the training group and 470 patients in the validation group. From a large sample and multicenter perspective, a multivariate survival analysis model was built, and a nomogram was drawn to elaborate the prognostic factors of malignant tumors of the uterine cervix in Asian women and analyze the relationship between them and survival. The AUC values of the training group at 3 and 5 years were 0.837 (95% CI: 0.790–0.882) and 0.818 (95%CI: 0.776–0.856), respectively, while the AUC values of the validation cohort at 3 and 5 years were 0.796 (95% CI: 0.731–0.858) and 0.783 (95% CI: 0.720–0.853), respectively, which suggested good model accuracy. In addition, some researchers hold the view that the calibration curve can judge whether the nomogram has a prediction error or overfitting. When the curve has a better fit to the 45° line, the prediction model is considered to have a better calibration ability^24^. Furthermore, the calibration curve of the nomogram in this study had a better fit with the 45° line and the higher AUC value reflected the better prediction precision of the model. Calibration curves showed that model consistency was acceptable, and K-M survival curves showed statistical significance for different ages and tumor sizes. To the best of our knowledge, this is the first study focusing on Asian CC patients based on the SEER database. In this study, age, AJCC stage, AJCC T, tumor size, and surgery were all independent predictors of OS in CC and were included in the nomogram.

Most CC prognostic nomograms include the traditional TNM stage, which shows that stage T3-4 has the worst prognosis than stage T1-2^15,16,18,19^. In addition, the older the patient is, the worse the prognosis is. In general, the older patients had a lower immune response, thus resulting in a poorer survival outcome, and our findings are also in agreement with this. The more advanced the clinical stage is, the lower the OS probability are. Because the residual lesions in patients in the early stages of AJCC staging are relatively small, they can be resected more completely at surgery. Better the response to chemotherapy, the lower the risk of recurrence and metastasis. Therefore, patients with these conditions have a good prognosis. The more extensive tumor cell spread in patients with advanced AJCC stages makes it difficult to implement radical debulking surgery, and chemotherapy is prone to drug resistance with worse prognosis. In 2018, according to FIGO staging of cervical cancer, patients with pelvic or para-aortic lymph node metastasis were classified as Stage IIIC1/2^25–27^. At present, many studies around the world proved that lymph node metastasis is one of the main prognostic indicators of cervical cancer, but this study has not clearly confirmed the actual impacts of this variable on the construction of the nomogram^15,16,18,19^, because this study defines the study population as Asian population with possible ethnic differences. In addition, LASSO regression was used to screen univariate variables in this study, which can minimize the collinearity of variables. Lymph node metastasis has been eliminated in univariate analysis. This study is an Asian population without limitation on stages. There are differences in the design of the study. We hold the view that such results do not conflict with previous studies. Therefore, after considering factors such as surgery and radiotherapy, whether the prognosis of lymph node metastasis is worse than that of stage I and stage II patients remains to be further studied. For a long time, the influences of histology on the prognosis of cervical cancer have been controversial^28^. Common pathological types of cervical cancer include cervical squamous cell carcinoma, adenocarcinoma, and other pathological types, including adenosquamous carcinoma, small cell neuroendocrine tumor of the cervix (NECC), clear cell carcinoma, sarcoma, etc. At present, although many scholars believe that the prognosis of patients with adenocarcinoma is poor, there is still controversy about the differences between the survival outcomes of patients with adenocarcinoma and squamous cell carcinoma. However, the results of this study preliminarily showed that there were no significant differences in survival outcomes between patients with adenocarcinoma and squamous cell carcinoma, which was consistent with the research of Pan et al.^29^. However, it is worth noting that the research object is not limited to staging, which may lead to some limitations in the results. A large-scale retrospective analysis study showed that the prognosis of patients with locally metastatic cervical cancer and adenocarcinoma was poor, while no significant survival differences were found between squamous carcinoma and adenocarcinoma in patients with distant metastatic cervical cancer, which may be related to artificial intervention methods, such as surgery, radiotherapy and chemotherapy, and risk factors, such as ovarian metastasis and vascular invasion. However, in patients with cervical cancer with distant metastasis, whether there is a difference in the prognosis between adenocarcinoma and squamous cell carcinoma still deserves attention^29^.

In terms of clinical treatment, more accurate substage stratification (FIGO or TNM) is really needed to identify patient subgroups at different prognostic levels, such as Phase IA, Phase IB, etc. However, this study considered that in clinical practice, the detailed FIGO or TNM staging may be inaccurate in some cases. Therefore, this study used a larger category of staging data as much as possible to facilitate the practical needs of nomography. Most importantly, as one of the most important prognostic indicators, the definition of “surgery” is related to the nursing standards used in any single geographical region. For example, in western countries, surgery is not usually used at a better stage than FIGO IB2 and IIA. It is believed that surgery is not an independent variable in the more favorable diffusion stage. There are many points to discuss about the impacts of the correlation between staging and surgery on the prognosis. If possible, this will be a new treatise.

Our nomographs are innovative and practical. Firstly, although the nomogram of cervical cancer has been widely used^15–19^, the nomogram of cervical cancer patients in Asia needs to be improved. Secondly, the nomogram can be used as a supplement to the FIGO staging system. The nomogram is simple to use, and clinical data such as age, AJCC staging, AJCC T, tumor size, and surgery can be easily obtained. Meanwhile, it can be used as a paper or online prediction tool to predict the prognosis of cervical cancer patients in Asia. What’s more, it can help clinicians distinguish patients who may benefit more from treatment, conduct clinical trials, and develop customized follow-up plans. Finally, the nomograph model also realizes the risk stratification of patients, which promotes personalized treatment plans and follow-up plans. For example, according to the AJCC staging system, the prognosis of AJCCIII-IV patients younger than 48 years old is poor. Therefore, patients may give up treatment due to financial burden. However, according to the nomogram of this study, the patients may have a better prognosis (OS > 50% in 3 years or even 5 years) to help clinicians make better strategic decisions to meet the needs of patients. In short, nomograms are helpful for clinical treatment and research of cervical cancer patients in Asia.

This study successfully constructed and validated a SEER database-based nomogram model for the prediction of survival in Asian CC patients, which contains indicators that are easily accessible with relatively accurate predictive ability, and the established risk stratification system also has some practical value. However, the following deficiencies remain: (1) the data from SEER database are retrospective analysis and draw on limited significance. (2) The data span is large and does not take the impacts on OS into account due to the advances in treatment options. (3) No further validation with external data is available, thereby pending further external validation with hospital or similar post hospital data.

### Electronic supplementary material

Below is the link to the electronic supplementary material.

Supplementary Figure 1.

Supplementary Figure 2.

Supplementary Figure 3.

Supplementary Table 1.

Supplementary Legends.

## Data Availability

The dataset for our study can be found in the Surveillance, Epidemiology, and End Results (SEER) database and Supplementary file.
