# Supplementary figures and images for: Development and validation of prognostic nomographs for patients with cervical cancer: SEER-based Asian population study
Source: Sci Rep. 2024 Apr 1;14:7681. doi: 10.1038/s41598-024-57609-7 (PMC10984919; doi:10.1038/s41598-024-57609-7)

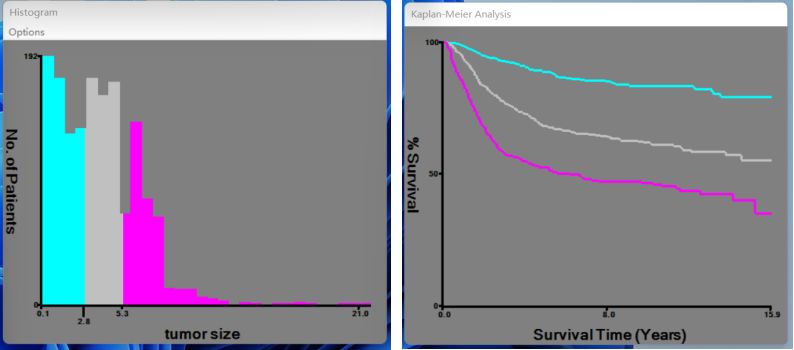

Supplement: Supplementary file 1 — Supplementary Figure 1. [file 41598_2024_57609_MOESM1_ESM.jpg]

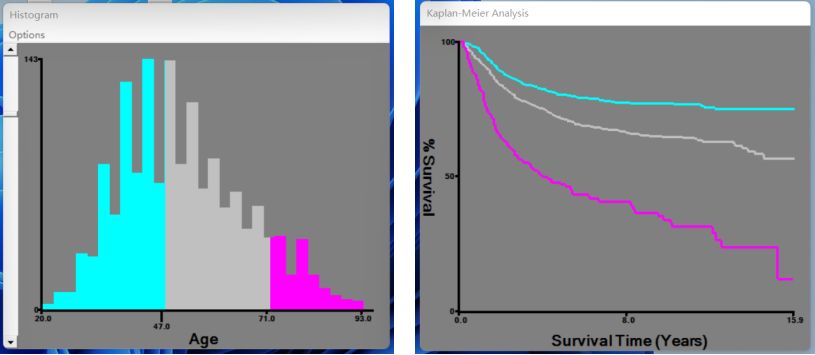

Supplement: Supplementary file 2 — Supplementary Figure 2. [file 41598_2024_57609_MOESM2_ESM.jpg]

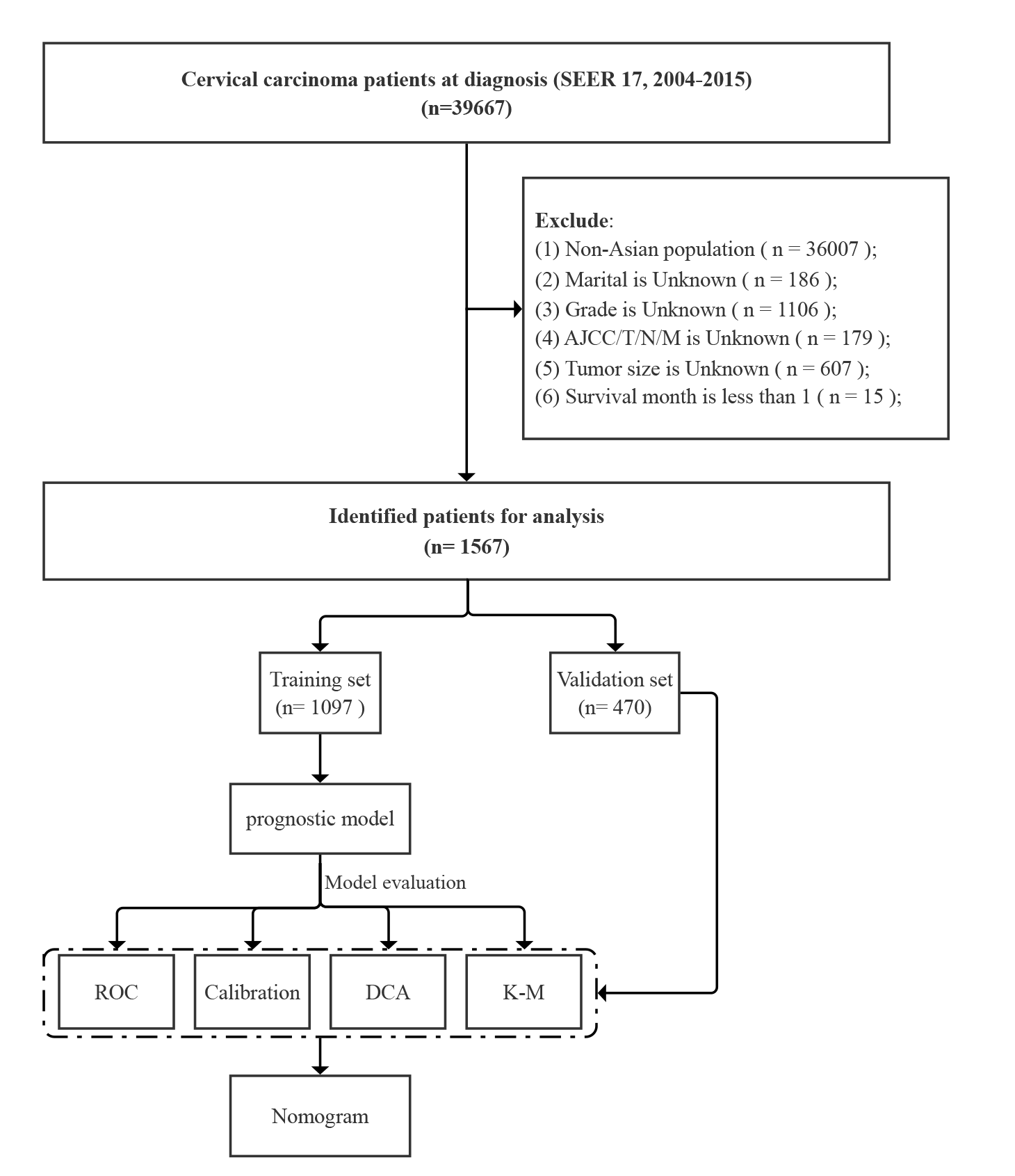

Supplement: Supplementary file 3 — Supplementary Figure 3. [file 41598_2024_57609_MOESM3_ESM.tif]
