# Supplementary material for: Development and validation of prognostic nomographs for patients with cervical cancer: SEER-based Asian population study
Source: Sci Rep. 2024 Apr 1;14:7681. doi: 10.1038/s41598-024-57609-7 (PMC10984919; doi:10.1038/s41598-024-57609-7)
Supplement: Supplementary file 5 — Supplementary Legends. [file 41598_2024_57609_MOESM5_ESM.docx]

[Supplementary](http://webvpn.dlut.edu.cn/https/77726476706e69737468656265737421e7e056d22122675e6a018cbe8b5c2d7b0ce92c/articles/10.3389/fonc.2021.742761/full#h12) Figure 1. Detailed data collection process.

[Supplementary](http://webvpn.dlut.edu.cn/https/77726476706e69737468656265737421e7e056d22122675e6a018cbe8b5c2d7b0ce92c/articles/10.3389/fonc.2021.742761/full#h12) Figure 2. Estimation of the cut-off value for the age determined by X-tile software.

[Supplementary](http://webvpn.dlut.edu.cn/https/77726476706e69737468656265737421e7e056d22122675e6a018cbe8b5c2d7b0ce92c/articles/10.3389/fonc.2021.742761/full#h12) Figure 3. Estimation of the cut-off value for the tumor size determined by X-tile software.

[Supplementary](http://webvpn.dlut.edu.cn/https/77726476706e69737468656265737421e7e056d22122675e6a018cbe8b5c2d7b0ce92c/articles/10.3389/fonc.2021.742761/full#h12) Table 1. The original data about patient information in this study.
